# Active-Assistive Control Based on Dynamic Moving Window for Trajectory Tracking of an Upper Limb Exoskeleton in Assisted Rehabilitation

**DOI:** 10.3390/s26072160

**Published:** 2026-03-31

**Authors:** Yuseop Sim, Jaehwan Kong, Seong-Sig Choi, Hak Yi

**Affiliations:** 1 School of Mechanical Engineering, Purdue University, 610 Purdue Mall, West Lafayette, IN 47907, USA; 2 Department of Mechanical Engineering, Kyungpook National University, 80 Daehak-ro, Buk-gu, Daegu 41566, Republic of Korea; korean1456@knu.ac.kr; 3 Daegu Center for Industrial Technology Convergence, Korea Institute of Industrial Technology, 320 Teokno-sunhwan-ro, Yuga-eup, Dalseong-gun, Daegu 42994, Republic of Korea

**Keywords:** exoskeleton, physical human–robot interaction, prosthetics, robotic rehabilitation

## Abstract

Rehabilitation robotics faces the challenges of aligning engineering design with patient-specific needs. Most existing controllers in rehabilitation robots often constrain motion to fixed paths or provide only passive guidance, limiting user engagement and adaptability. This study proposes a novel active-assistive mode controller that integrates a virtual tunnel-based force generation mechanism with a dynamic moving-window technique for tracking activities of daily living (ADL) trajectories. Unlike conventional impedance controllers, the proposed method dynamically adjusts the virtual tunnel in real time, permitting voluntary upper-limb movement within a safe operational range while preventing excessive deviation. The system was implemented on a wearable two-degree-of-freedom (DOF) upper-limb exoskeleton equipped with drive and integrated sensor units. Experimental results demonstrated that decreasing the guidance force ($F_{gf}$) increased tracking errors, from 1° at 100% $F_{gf}$ to 5° at 30% $F_{gf}$, indicating greater voluntary participant motion. Peak actuator torques correspondingly decreased from 14.75 to 13.43 Nm (elbow) and from 4.14 to 2.48 Nm (wrist), confirming the controller’s capability to modulate robotic assistance according to user effort. Tests with 30 healthy participants demonstrated the effectiveness of guidance along predefined ADL trajectories, validating the controller’s potential for patient-centered rehabilitation.

## 1. Introduction

Exoskeleton robots have gained recent prominence in the rehabilitation of patients with motor impairments [1,2,3,4]. When designed to be lightweight and wearable, these exoskeletons can be tailored to individuals’ rehabilitation needs. Such patient-centered wearable robots facilitate task-oriented rehabilitation exercises in unconstrained environments, supporting the restoration of activities of daily living (ADL) [5,6]. For instance, a versatile and dynamic upper-limb rehabilitation robot incorporates 15 distinct ADL movements into stroke rehabilitation training [7].

Recent research has garnered significant interest in regulating robotic-assisted rehabilitation to accommodate the individual physical needs of each patient [8,9,10]. Such systems offer multidimensional stimulation that fosters motor recovery and cognitive and sensory engagement. A key strategy is the active-assistive mode, in which the robot provides partial support when patients cannot execute movements independently. Chen et al. [8] explored an assist-as-needed control scheme to guide stroke patients along predefined exercise trajectories. Chungunang et al. [9] implemented impedance control for active-assisted and active-resisted single-elbow movement training, highlighting its potential to enhance bimanual training through upper limb rehabilitation robots. Ugurlu et al. [10] developed a torque controller using a disturbance observer to generate and transmit assistive forces to users. Conventionally, robot-assisted rehabilitation has relied on large-scale, end-effector-type systems. However, growing emphasis on ADL-oriented rehabilitation has redirected research toward advanced exoskeleton control strategies that better emulate a therapist’s intervention and adapt sensitively to individual conditions.

To effectively mimic therapist movements and enhance rehabilitation outcomes, it is important to account for uncertainties in patient behavior, including variations in participation and muscle activation [11]. To address these challenges, various control strategies, such as adaptive fuzzy systems, have been developed to compensate for such uncertainties in human–robot interaction [12]. Incorporating therapist-motion imitation allows robots to deliver more natural and personalized training that supports motor learning through consistent yet human-like guidance [13]. Consequently, an adaptive controller that accommodates patient-specific variability while maintaining predefined motion trajectories is crucial for effective rehabilitation.

Table 1 summarizes representative control-oriented studies on upper-limb rehabilitation exoskeletons and related platforms that align with the scope of this work (active-assistive trajectory tracking and safe guidance). From a control perspective, prior studies have addressed trajectory tracking, impedance/admittance-based assistance, and assist-as-needed schemes that modulate assistance according to user engagement, often combined with allowable-region guidance (e.g., virtual fixtures/tunnels or field-based guidance) to preserve user initiative. In particular, the present work builds on these control concepts by introducing a moving-window guidance mechanism that maintains an allowable region while adapting the guidance force in real time.

Building on these developments, numerous upper-limb rehabilitation robots have been designed to address patient-specific needs (Table 1) [14,15,16,17,18,19,20]. Over the past decade, the growing emphasis on ADL-based rehabilitation has intensified interest in exoskeleton robots equipped with advanced control strategies [3,14,15]. Most studies on robot-assisted recovery focus on precise regulation of interaction forces along predefined exercise trajectories to enhance performance and ensure patient safety. Maintaining stable and smooth motion is critical, particularly for individuals with restricted joint mobility. To address these challenges, force-field-based control techniques have been proposed to guide joint movement within a compliant region around the reference trajectory, commonly termed a virtual tunnel. Over the past three years (2023–2026), assist-as-needed control has been further extended while retaining the virtual-tunnel/field-based allowable-region concept, by incorporating performance-adaptive assistance (including timing freedom) and variable admittance/impedance modulation [21,22,23]. A representative implementation is the moving-window method [24], which enhances robot motion stability during rehabilitation. Duschau-Wicke et al. [25] demonstrated that a moving-window controller effectively guided patients along predefined ambulation paths, while Luo et al. [26] reported improved tracking performance in a simplified wrist rehabilitation system. Similarly, Bucchieri et al. [27] employed virtual-tunnel control to direct upper-limb movements in pick-and-place tasks, achieving smooth and safe motion within the permitted workspace.

Despite significant progress in wearable rehabilitation robotics, active-assisted force control that simultaneously supports basic upper-limb exercises and enforces safe, functional ranges remains limited. To bridge this gap, this study integrates a moving-window approach with a virtual tunnel-based active-assistive control system for a two-degree-of-freedom (2-DOF) wearable upper-limb exoskeleton. Unlike conventional rigid boundary controllers, the proposed framework dynamically modulates assistive forces in real time according to the user’s capabilities, enabling adaptive, patient-centered rehabilitation. Elbow and wrist assistive torques are computed through a virtual impedance model, promoting voluntary yet safely constrained motion during rehabilitation.

## 2. System Overview

**Figure 1:**
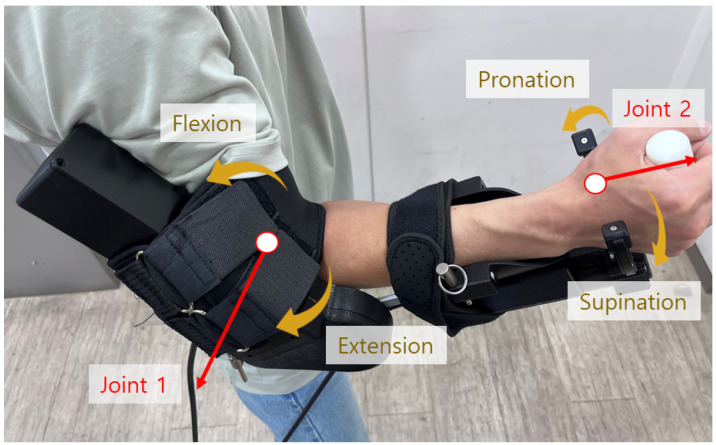
Robot joints and human motions.

The proposed upper-limb exoskeleton is developed to assist human arm movements during ADL (Figure 1). The system comprises three modules: (1) mechanical structure and actuators, (2) sensing and data acquisition units, and (3) the control module.

### 2.1. Forearm/Arm Exoskeleton Description

The forearm–arm exoskeleton was designed to fit the anthropometric profile of adult Asian males, covering 25–75% of average arm length based on statistical human body data [28]. The flexion–extension of the right elbow and pronation–supination of the right wrist are realized through revolute joints. The frame housing the drive units and sensors is constructed from aluminum 6061, while the outer casing uses acrylonitrile–butadiene–styrene for electrical insulation and lightweight durability. The exoskeleton weighs 2.0 kg, with an additional 0.25 kg from cuffs and bands to secure the arm during rehabilitation exercises.

Each joint is actuated by a brushless DC (BLDC) motor (EC45 Flat, maxon, Sachseln, Switzerland). The elbow joint’s motor employs a 1:18 gear train, and the torque is further increased through a belt-pulley mechanism with an additional 1:5.88 ratio, for a total gear ratio of 1:105.84. The wrist joint’s motor uses a planetary gear set (1:9.67 ratio) with an extra 1:2.31 ratio between the planetary carrier and the supporting gear, yielding a total gear ratio of 1:22.34. Accordingly, the elbow and wrist actuators produce nominal output torques of 12.53 Nm and 3.57 Nm, respectively, sufficient for standard rehabilitation exercises [29].

Each module includes an STM32H743 microcontroller (STMicroelectronics, Plan-les-Ouates, Geneva, Switzerland) to drive the motor and transmit sensor data to the main PC. The collected data are consolidated into a single packet to facilitate human–robot interaction during rehabilitation training, including real-time feedback to the user. Two cables handle communication protocols between the peripheral devices: the motor controller (EPOS2 70/10, maxon, Sachseln, Switzerland) communicates via RS-232, while the six-axis force/torque sensor (RFT82, Robotous, Seongnam-si, Gyeonggi-do, Republic of Korea), mounted at the distal end of the robot, communicates via RS-422. All control loops operate at 100 Hz.

### 2.2. Kinematic Model

**Figure 2:**
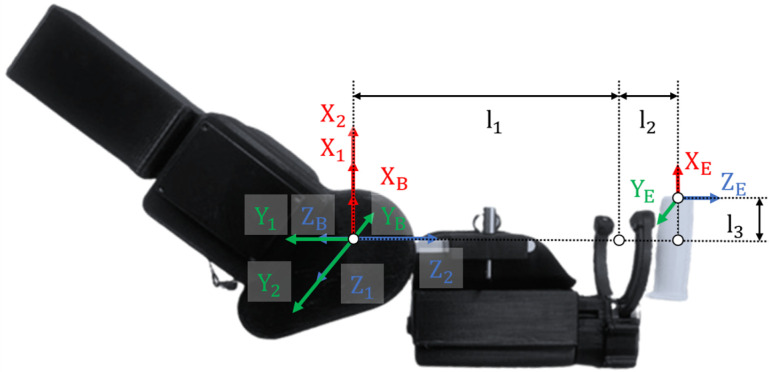
Kinematic arrangement of axes.

Figure 2 and Table 2 display the exoskeleton robot’s coordinate system and the Denavit–Hartenberg parameters, respectively. These parameters include $a_{i-1}$ (link length), $\alpha_{i-1}$ (link twist), $d_{i}$ (link offset), and $\theta_{i}$ (joint angle). In addition, $\theta_{1}$ and $\theta_{2}$ denote the rotational angle of the elbow and wrist joints, respectively. The position of the robot end-effector and its Jacobian matrix are expressed in Equations (1) and (2), respectively.

(1) $$\begin{array}{cc} P\;=\; & {\left[\begin{array}{ccc} l_{1+2}s_{1}+l_{3}c_{1}c_{2} & -l_{3}s_{2} & l_{3}c_{2}s_{1}-l_{1+2}c_{1} \end{array}\right]}^{T} \end{array}$$

(2) $$J\;=\;{\left[\begin{array}{ccc} l_{1+2}c_{1}\;-\;l_{3}s_{1} & 0 & l_{1+2}s_{1}\;+\;l_{3}c_{1+2}\\ -l_{3}c_{1}s_{2} & -l_{3}c_{2} & -l_{3}s_{1}s_{2} \end{array}\right]}^{T}$$

where $s_{i}\;=\;\sin\left(\theta_{i}\right)$, $s_{j}\;=\;\sin\left(\theta_{j}\right)$, $c_{i}\;=\;\cos\left(\theta_{i}\right)$, $c_{j}\;=\;\cos\left(\theta_{j}\right)$, $l_{i+j}\;=\;l_{i}+l_{j}$, and $c_{i+j}\;=\;\cos\left(\theta_{i}+\theta_{j}\right)$.

### 2.3. Dynamic Model

The exoskeleton’s dynamic behavior, derived from the Lagrangian formulation, is expressed in Equation (3). Each joint is modeled considering its corresponding motor, transmission, and link inertia. The inertia tensors of the first and second links, $I_{1}$ and $I_{2}$, are defined with respect to their centers of mass coordinate frames. Each tensor comprises principal moments of inertia ($I_{ixx}$, $I_{iyy}$, and $I_{izz}$) along the *x*-, *y*-, and *z*-axes, and products of inertia ($I_{ixy}$, $I_{ixz}$, and $I_{iyz}$) representing the coupling between the respective axes (i=1, 2).

(3) $$\begin{array}{c} \tau \;=\;M\left(\theta \right)\ddot{\theta }\;+\;C\left(\theta ,\dot{\theta }\right)\dot{\theta }\;+\;g\left(\theta \right);\\ M\left(\theta \right)\;=\;\left[\begin{array}{cc} m_{11} & I_{2yz}c_{2}\;+\;I_{2xz}s_{2}\\ I_{2yz}c_{2}\;+\;I_{2xz}s_{2} & I_{2zz} \end{array}\right],\\ C\left(\theta ,\dot{\theta }\right)\;=\;\left[\begin{array}{cc} {\dot{\theta }}_{2}\alpha & {\dot{\theta }}_{1}\alpha \\ {\dot{\theta }}_{1}\alpha \;+\;{\dot{\theta }}_{2}\left(I_{2xz}c_{2}\;-\;I_{2yz}s_{2}\right) & 0 \end{array}\right],\\ g\left(\theta \right)\;=\;{\left[\begin{array}{cc} gs_{1}\left(m_{1}l_{1}\;+\;m_{2}l_{2}\right) & 0 \end{array}\right]}^{T} \end{array}$$

where $m_{11}\;=\;\left(I_{1yy}\;+\;I_{2yy}\right)c_{2}^{2}\;+\;\left(I_{1xx}\;+\;I_{2xx}\right)s_{2}^{2}\;+\;m_{2}l_{1\;+\;2}^{2}\;+\;2\left(I_{1xy}\;+\;I_{2xy}\right)s_{2}c_{2}$; $\alpha \;=\;\left(1\;-\;2s_{2}^{2}\right)\left(I_{1xy}\;+\;I_{2xy}\right)$; M(θ) and $C(\theta ,\dot{\theta })$ signify the inertia matrix and Coriolis/centrifugal forces, respectively; g(θ) denotes the gravity vector; τ represents the total joint torque acting on each joint; $m_{1}$ and $m_{2}$ correspond to the masses of the forearm-to-wrist and the wrist-to-handle modules, respectively.

## 3. Moving Window-Based Control

### 3.1. Methodology

**Figure 3:**
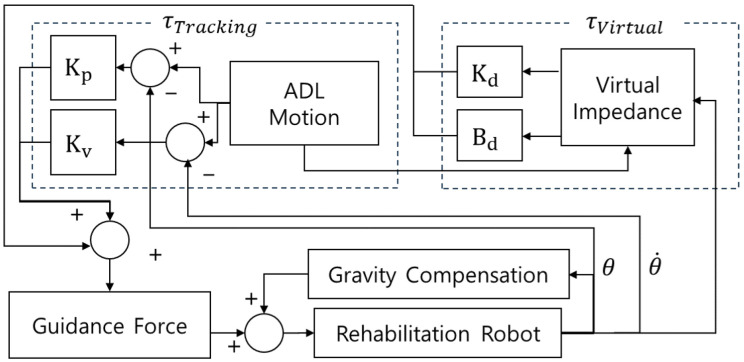
Block diagram of the proposed controller.

This study proposes a dynamic moving spatial window, centered on each joint’s reference trajectory, to regulate the exoskeleton’s active-assistive behavior. By establishing an adaptive spatial boundary around the reference trajectory, the system adjusts assistance or resistance based on user effort, providing intuitive motion guidance during rehabilitation. Within the boundary, the active-assistive mode enables patients to move more freely and independently [27,30]. The moving window is realized using a combination of virtual tunnel and back-wall concepts. The back wall acts as a virtual impedance controller, limiting excessive motion and enforcing trajectory tracking within the defined region (Figure 3). When the patient requires a generated torque to follow the reference trajectory, the system delivers guidance-force assistance, modulated by the guidance-force parameter and passive-mode torque. If motion exceeds the reference window, the system applies assisted resistance to restore the limb to the desired range of motion. Substituting $\tau \;=\;\tau_{control}\;-\;\tau_{ext}$ into Equation (3), where $\tau_{control}$ and $\tau_{ext}$ denote the actuator command and external disturbance, respectively, yields

(4) $$\ddot{\theta }\;=\;M{\left(\theta \right)}^{-1}\left(\tau_{control}\;-\;\tau_{ext}\;-\;C\left(\theta ,\dot{\theta }\right)\dot{\theta }\;-\;g\left(\theta \right)\right)$$

The control input, *u*, is defined in terms of $\ddot{e}$ as follows

(5) $$u\;=\;\ddot{e}\;=\;\ddot{\theta_{d}}\;-\;\ddot{\theta }$$

The control torque can be expressed as

(6) $$\tau_{control}\;=\;M\left(\theta \right)\left(\ddot{\theta_{d}}\;-\;u\right)\;+\;C\left(\theta ,\dot{\theta }\right)\dot{\theta }\;+\;g\left(\theta \right)\;+\;\tau_{ext}$$

To track the desired trajectory and enforce the virtual-wall constraint, the acceleration input, *u*, is designed as

(7) $$u\;=\;-F_{gf}M{\left(\theta \right)}^{-1}\left(\tau_{tracking}\;+\;\tau_{virtual}\right)$$

where $\tau_{tracking}$ denotes the feedback torque that minimizes the joint tracking error, and $\tau_{virtual}$ represents the boundary-enforcement torque from the moving window (virtual wall) that drives and damps motion back into the allowable region.

The guidance force, $F_{gf}$, in rehabilitation control mode can be adjusted between 0% and 100% of the supportive force. At 100%, the system provides full supportive force to maintain the targeted joint angle, even when the patient is unable to move [31]. When $F_{gf}$ is set below 100%, the robot operates in an active-assistive mode, supporting partial support to complement the patient’s voluntary movement. Nevertheless, due to the difficulty of obtaining precise measurement data, maintaining movement within a defined range of the desired trajectory is often more effective than enforcing exact tracking. At 0%, the robot only compensates for gravity, enabling fully voluntary movement. Substituting the input *u* into Equation (6) yields

(8) $$\begin{array}{cc} \tau_{\mathrm{control}}\;=\; & M\left(\theta \right){\ddot{\theta }}_{d}\;+\;F_{gf}\left(\tau_{tracking}\;+\;\tau_{\mathrm{virtual}}\right)\\ & +\;C\left(\theta ,\dot{\theta }\right)\dot{\theta }\;+\;g\left(\theta \right)\;+\;\tau_{\mathrm{ext}} \end{array}$$

where the Coriolis and centrifugal terms are omitted from the control formulation due to the lightweight structure of the upper-limb robot, low operating speed, and the influence of friction during slow rehabilitation motions. Under quasi-static conditions with limited joint acceleration, the Coriolis effects remain bounded and exert minimal impact on system stability. Therefore, excluding these terms is a valid simplification of the Euler–Lagrange-based dynamic model for this operating regime. Considering only the dominant quasi-static terms, Equation (8) reduces to

(9) $$\tau_{\mathrm{control}}\;\approx \;F_{gf}\left(\tau_{\mathrm{virtual}}+\;\tau_{\mathrm{tracking}}\right)\;+\;g(\theta )$$

where $\tau_{virtual}$ and $\tau_{tracking}$ denote the control torques based on virtual impedance and trajectory tracking, respectively; g(θ) signifies the gravity compensation term that eliminates any effect of the robot’s weight. The guidance force adjusts the characteristics of active-assistive mode exercises.

Impedance control, first proposed by Hogan [32], defines the mapping relationship between input motion (displacement and its derivatives) and resulting forces or torques. In this study, a virtual impedance model, characterized by virtual stiffness, $K_{d}$, and damping, $B_{d}$, represents the interaction between each joint angle and the upper and lower limits of a moving window that forms a constant-sized square around the defined trajectory. These gains, determined in consultation with rehabilitation therapists, generate repulsive torques tailored to the patient’s condition. In the experiments, $K_{d}\;=\;\mathrm{diag}\left[0.1,\;0.1\right]$, $B_{d}\;=\;\mathrm{diag}\left[0.001,\;0.001\right]$, and $w_{\mathrm{depth}}\;=\;5{}^{\circ}$ (corresponding to a 10° moving window).

(10) $$\tau_{virtual}\;=\;B_{d}{\dot{d}}_{vir}\;+\;K_{d}d_{vir}$$

(11) $$d_{vir}\;=\;\left\{\begin{array}{cc} \frac{w_{\mathrm{depth}}\left(\theta \;-\;\theta_{d}\right)}{\theta_{\mathrm{upper}}\;-\;\theta }, & \mathrm{for}\;\theta_{d}\;<\;\theta \;<\;\theta_{\mathrm{upper}},\\ \frac{w_{\mathrm{depth}}\left(\theta \;-\;\theta_{d}\right)}{\theta \;-\;\theta_{\mathrm{lower}}}, & \mathrm{for}\;\theta_{\mathrm{lower}}\;<\;\theta \;<\;\theta_{d}. \end{array}\right.$$

where $B_{d}$ and $K_{d}$ denote 2 × 2 diagonal matrices; $d_{vir}$ signifies the virtual displacement of repulsion between the current joint angle and the upper and lower bounds of the moving window (Equation (11)); $\theta_{upper}$ and $\theta_{lower}$ represent the upper and bottom limits of the moving window, respectively; $w_{depth}$ refers to the depth (safety region) on each boundary of the virtual wall; and $\theta_{d}$ specifies the targeted joint angle along the predefined trajectory.

A proportional-derivative (PD) controller was implemented to track the predefined trajectory. Specifically, the gains used in the experiments were $K_{p}\;=\;\mathrm{diag}\left[50,000,\;30,000\right]$ and $K_{v}\;=\;\mathrm{diag}\left[100,\;55\right]$ for the elbow and wrist joints.

(12) $$\tau_{tracking}\;=\;K_{v}\left({\dot{\theta }}_{d}\;-\;\dot{\theta }\right)\;+\;K_{p}\left(\theta_{d}\;-\;\theta \right)$$

Gravity compensation was implemented to alleviate the effects due to the robot’s weight as

(13) $$g\left(\theta \right)\;=\;{\left[gs_{1}\left(m_{1}l_{1}\;+\;m_{2}l_{2}\right)\;0\right]}^{T}$$

The gravity compensation torque was applied to the robot as a feed-forward term, reaching its maximum when the lower arm, initially oriented downward, formed a right angle with the upper arm.

### 3.2. Stability Analysis

The Lyapunov function, augmented with the elastic energy of the virtual wall, is defined as

(14) $$V\;=\;\frac{1}{2}{\dot{e}}^{⊤}M\left(\theta \right)\dot{e}\;+\;\frac{1}{2}F_{gf}\;\left(e^{⊤}K_{p}e\;+\;d_{\mathrm{vir}}^{⊤}K_{d}d_{\mathrm{vir}}\right)$$

where $F_{gf}\;\in \;\left[0,\;1\right]$, $K_{p}≻0$, and $K_{d}≻0$. The time derivative of *V* can be expressed as

(15) $$\dot{V}\;=\;{\dot{e}}^{⊤}M\left(\theta \right)\ddot{e}\;+\;F_{gf}\left({\dot{e}}^{⊤}K_{p}e\;+\;{\dot{d}}_{\mathrm{vir}}^{⊤}K_{d}d_{\mathrm{vir}}\right)$$

By substituting Equations (3), (8), (10) and (12) into (15), the derivative of the Lyapunov function can be simplified as

(16) $$\dot{V}\;=\;F_{gf}\left(-{\dot{e}}^{⊤}\left[K_{v}\dot{e}\;+\;K_{d}d_{\mathrm{vir}}\;+\;B_{d}{\dot{d}}_{\mathrm{vir}}\right]\;+\;{\dot{d}}_{\mathrm{vir}}^{⊤}K_{d}d_{\mathrm{vir}}\right)$$

System stability is ensured when the proportional damping term ($K_{v}$) outweighs the potentially destabilizing virtual damping term ($-{\dot{e}}^{⊤}B_{d}{\dot{d}}_{\mathrm{vir}}$). Applying the chain rule,

(17) $${\dot{d}}_{\mathrm{vir}}\;=\;\frac{\partial d_{\mathrm{vir}}}{\partial \theta }\frac{d\theta }{dt}\;=\;\Lambda \dot{\theta }\;=\;\Lambda \left({\dot{\theta }}_{d}\;-\;\dot{e}\right)$$

Substituting Equation (17) into the virtual damping yields the net energy damping term $E_{\mathrm{Net}}$:

(18) $$E_{\mathrm{Net}}\;=\;-F_{gf}{\dot{e}}^{⊤}\left(K_{v}\;-\;B_{d}\Lambda \right)\dot{e}\underset{\mathrm{Residual}\;\Delta_{1}}{\underset{︸}{\;-\;F_{gf}{\dot{e}}^{⊤}B_{d}\Lambda {\dot{\theta }}_{d}}}$$

To guarantee energy dissipation ($E_{\mathrm{Net}}\;-\;\Delta_{1}\;\leq \;0$), the unbounded term Λ near the joint limits is clipped to a bounded value, denoted by $\Lambda_{\mathrm{clipped}}$. This ensures that the dissipation matrix $P\;=\;K_{v}\;-\;B_{d}\Lambda_{\mathrm{clipped}}$ remains positive definite (P≻0). The corresponding upper bound, $\Lambda_{\max}$, is defined as

(19) $$\Lambda_{\mathrm{clipped}}\;=\;\min\left(\Lambda ,\Lambda_{\max}\right),\;\mathrm{with}\;\Lambda_{\max}≺B_{d}^{-1}K_{v}$$

Finally, by applying the Λ clipping, $\dot{V}$ can be expressed as the sum of a guaranteed dissipative term and a bounded residual term associated with $\Delta_{1}$:

(20) $$\begin{array}{cc} \dot{V}\;\leq \; & -F_{gf}\;{\dot{e}}^{⊤}P\dot{e}\\ \; & +\;F_{gf}\;\underset{\mathrm{Bounded}\;\mathrm{Residual}\;\mathrm{Term}\;\Delta_{\mathrm{UUB}}}{\underset{︸}{\left[\left(d_{\mathrm{vir}}^{⊤}K_{d}{\dot{d}}_{\mathrm{vir}}\;-\;{\dot{e}}^{⊤}K_{d}d_{\mathrm{vir}}\right)\;-\;{\dot{e}}^{⊤}B_{d}\Lambda_{\mathrm{clipped}}{\dot{\theta }}_{d}\right]}} \end{array}$$

Therefore, the system attains uniform ultimate boundedness (UUB), with the tracking error remaining constrained. Moreover, $\dot{V}\;<\;0$ holds when the velocity error $∥\dot{e}∥$ exceeds the threshold determined by the energy balance, scaled by the minimum eigenvalue $\lambda_{\min}\left(P\right)$:

(21) $$\mathrm{If}\;∥\dot{e}∥\;>\;\frac{∥\Delta_{\mathrm{UUB}}∥}{\lambda_{\min}\left(P\right)},\;\mathrm{then}\;\dot{V}\;<\;0$$

## 4. Experiments

The control algorithm (Section 3) was evaluated using two representative ADLs: hand washing and door opening. These tasks capture essential upper-limb motions: wrist pronation–supination and elbow flexion–extension, fundamental for independent self-care [33]. The door-opening task assesses the controller’s ability to generate sufficient assistive torque during elbow flexion and wrist pronation, while the hand-washing task evaluates coordination during repetitive wrist rotations with a nearly static forearm. These tasks provide complementary motion patterns that serve as practical benchmarks for the proposed moving-window-based control strategy. Motion-capture trajectories used for reference ADL trajectory generation were obtained from all participants (N = 30), and the resulting trajectories were used to define the ROM envelope employed in the experiments.

### 4.1. Data-Driven Trajectory Generation

**Figure 4:**
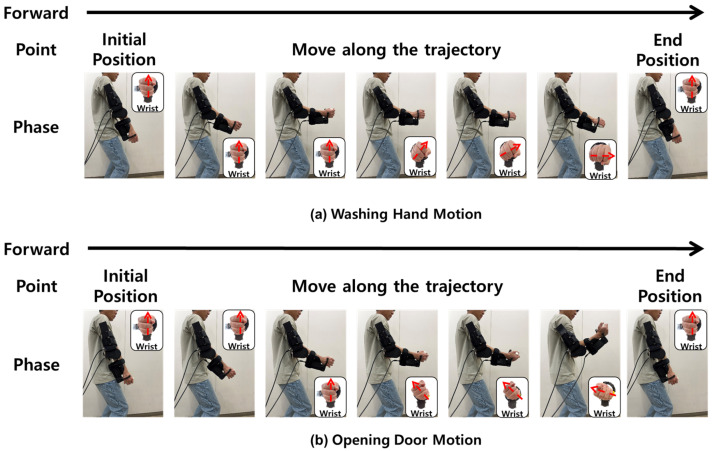
Step-by-step snapshots of the ADL tasks: (**a**) hand-washing motion from the initial position to the goal position and back; (**b**) door-opening motion from the initial position to the goal position and back. The assisted wrist DOF is highlighted with inset views and arrows.

A cohort of 30 healthy participants (Table 3), with a mean age of 27.8 ± 5.4 years, completed evaluations of two ADL motions using the proposed two-DOF robotic device. As illustrated in Figure 4, each motion followed a 15 s cycle within specific joint angle ranges: −50° to 50° for the wrist and −20° to 120° for the elbow. Each participant generated a reference trajectory by moving from a neutral posture, executing the motion, and returning to neutral within one cycle. Motion-capture trajectories used for reference ADL trajectory generation were obtained from all participants (N = 30).

**Figure 5:**
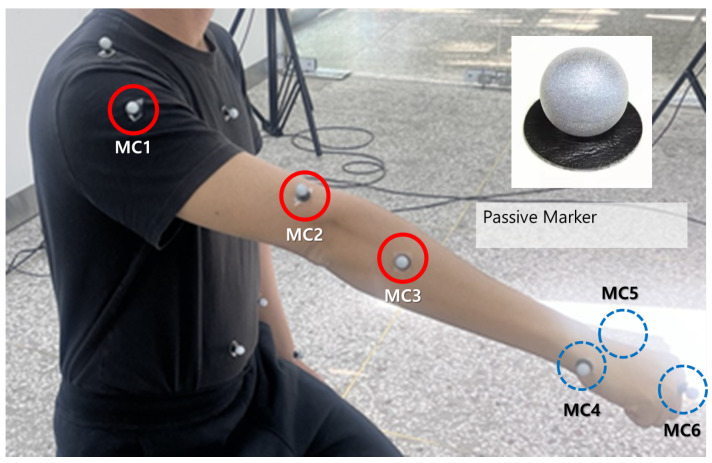
OptiTrack passive marker placement on the upper limb (MC1–MC6): upper arm (MC1–MC3) and forearm/hand (MC4–MC6).

Motion capture data were collected using an OptiTrack system (Flex13, NaturalPoint Inc. DBA OptiTrack, Corvallis, OR, USA; six cameras, 120 Hz; ±0.2 mm spatial accuracy). Six reflective markers were strategically attached to the participants’ right upper limbs to measure elbow and wrist joint rotations (Figure 5). The cameras were evenly distributed around the participant to cover the upper-limb workspace, and 3D marker positions were reconstructed through camera calibration. The resulting data were interpolated using polynomial regression and smoothed with a Savitzky–Golay filter to minimize signal distortion. The polynomial degree was determined by increasing the order until further increases yielded only marginal reduction in fitting error (RMSE), and the lowest order satisfying this criterion was used. This process yielded smooth and reliable reference trajectories for subsequent analysis [34].

**Figure 6:**
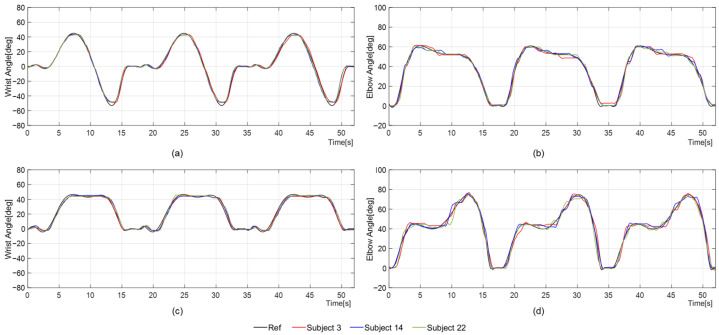
Passive mode training: (**a**) wrist and (**b**) elbow joints during the hand-washing motion; (**c**) wrist and (**d**) elbow joints during the door-opening motion.

Arm motions were recorded over a fixed duration to derive reference trajectories for the two ADL tasks, depicted as solid lines in Figure 6. For the hand-washing task, the elbow and wrist trajectories were modeled with 8th- and 9th-degree polynomials ($R^{2}\;=\;0.8857$; $R^{2}\;=\;0.8520$), respectively. Participants raised their arms toward the sink for 4 s, performed 14 s of wrist pronation–supination to simulate washing, and then the robotic arm returned to its initial position. For the door-opening task, the elbow and wrist trajectories converged at the 6th and 8th degrees ($R^{2}\;=\;0.9002$; $R^{2}\;=\;0.9156$), respectively. Participants lifted their arms to the door handle within 4 s, rotated their wrists for 4–6 s to turn it, pulled the door open over the next 10 s using elbow joints, and then returned to the starting posture at 14 s. Among the 30 healthy participants, door-opening motions were more consistent, as reflected by higher $R^{2}$ values, whereas wrist movements during the hand-washing task exhibited greater variability, leading to noticeable deviations from the reference trajectory. A participant-level summary of the ROM-based trajectory generation results from all 30 participants is provided in Appendix A Table A1.

### 4.2. Passive Mode Control

To evaluate the robot’s performance, passive-mode exercises were conducted with three repetitions of each ADL task: hand-washing and door-opening, within the predefined ranges of motion (ROM): −50° to 50° for the wrist and −20° to 120° for the elbow. Each repetition corresponded to one 15 s cycle defined in Section 4.1, resulting in a total duration of 45 s per task. A short rest was allowed between repetitions as needed. Thirty healthy participants performed these passive mode trials. Figure 6a–d show the rotational angle changes of wrist pronation/supination and elbow flexion/extension for three representative participants, representing the average, closest, and furthest tracking behaviors relative to the reference trajectory. While individual physical differences caused minor variations in trajectory tracking, the robotic joints consistently reached the target positions with minimal error. The root mean square (RMS) tracking errors for the wrist and elbow were 3.80° and 2.93°, respectively, during the hand-washing task, and 2.40° and 2.14° during the door-opening motion. These findings validate that the proposed passive control method achieves reliable trajectory tracking, maintaining RMS errors below 4°. This level of joint-angle tracking error (RMS <4°) is within the range reported in prior upper-limb rehabilitation exoskeleton studies under trajectory-tracking conditions [35]. A participant-level summary of the passive-mode experimental results from a representative subset of 15 participants is provided in Appendix A Table A2.

### 4.3. Active-Assistive Control with a Moving Window

Active-assisted mode training for the two ADL tasks was experimentally tested over 45 s with three repetitions. This corresponds to three consecutive 15 s cycles per task, consistent with the reference-trajectory definition in Section 4.1. A ±5° moving window around the target angle was defined to facilitate participants’ active movement. In all cases, $F_{gf}$ was set at three distinct levels (100%, 70%, and 30%) to generate assistive torque. Compared to passive-mode training, the active-assisted mode allowed participants to perform independent, voluntary motions within the designated window.

**Figure 7:**
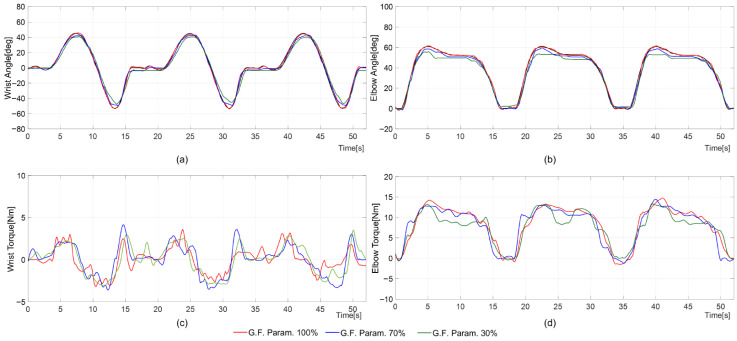
Reference (black line) and measured trajectories (red, blue, and green lines) in active-assistive mode rehabilitation training during the hand-washing motion with different guidance force parameters (red: subject 3, blue: subject 14, green: subject 22): (**a**) wrist angle position; (**b**) elbow angle position; (**c**) wrist motor-generated torque; and (**d**) elbow motor-generated torque. Note: The time–response plots show representative tracking examples.

**Figure 8:**
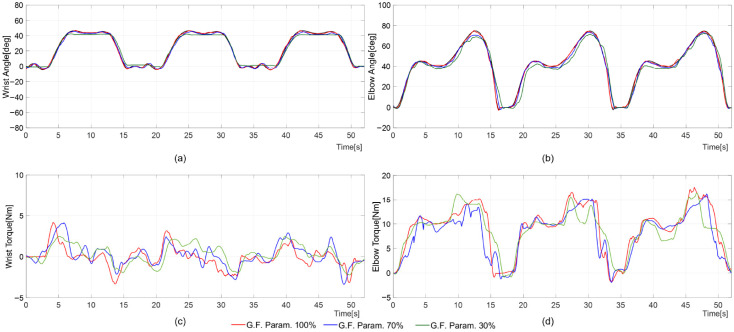
Reference (black line) and measured trajectories (red, blue, and green lines) in active-assistive mode rehabilitation training during the door-opening motion with different guidance force parameters (red: subject 3, blue: subject 14, green: subject 22): (**a**) wrist angle position; (**b**) elbow angle position; (**c**) wrist motor-generated torque; and (**d**) elbow motor-generated torque. Note: The time–response plots show representative tracking examples.

Figure 7 and Figure 8 are provided as representative time–response examples to illustrate the tracking behavior under different assistance conditions, while the corresponding tracking errors are summarized in Table 4 (mean ± SD) over the evaluated trials. Group-level tracking errors are reported as mean ± standard deviation across participants for each assistance condition (Table 4).

In the representative time–response examples shown in Figure 7 and Figure 8, at 30% $F_{gf}$, users voluntarily move their arm to follow the reference trajectory, producing only minimal differences in wrist/elbow angle changes during ADL movements compared to 100% or 70% $F_{gf}$. During the hand-washing motion, the wrist’s mean and standard deviation of the tracking error were ∼1.58±1.58° at 100% $F_{gf}$, increasing to 2.57±2.54° at 70% $F_{gf}$ and 4.85±4.79° at 30% $F_{gf}$. Similarly, the elbow’s mean and standard deviation of the tracking error rose from 1.21±1.17° at 100% to 2.45±1.82° at 70% and 4.09±3.01° at 30% $F_{gf}$. For the door-opening motion, the wrist’s mean and standard deviation of the tracking error were 0.96±0.96°, 1.73±1.61°, and 3.03±2.99°, while the elbow’s was 0.96±0.88°, 2.47±2.29°, and 4.81±4.44° at 100%, 70%, and 30%, respectively. Notably, at higher $F_{gf}$ levels, the active-assistive mode training control resembled passive mode training. During the motion, the motor-generated assistive torque varied with changes in $F_{gf}$. At 30% $F_{gf}$, the measured torque on each joint was relatively smaller than at higher levels. In particular, the peak torque consistently decreased as $F_{gf}$ reduced (Figure 8). For example, during the hand-washing task, the elbow motor produced peak torques of 14.75 Nm, 14.45 Nm, and 13.43 Nm, at 100%, 70%, and 30% $F_{gf}$, respectively. Similarly, during the door-opening motion, the wrist motor generated peak torques of 4.14 Nm, 4.10 Nm, and 2.48 Nm, at 100%, 70%, and 30% $F_{gf}$, respectively. In the active-assistive control mode, both the wrist and elbow joints exhibited stable performance throughout all training sessions. Regardless of the $F_{gf}$ level, the overall trends in joint range of motion (ROM) and supporting torque remained consistent, with only minor variations in tracking accuracy. As $F_{gf}$ decreased, participants contributed more actively to perform the movement, and the corresponding reduction in assistive torque increased deviations from the reference trajectory. At lower $F_{gf}$ values, the robots relied more heavily on the participants’ self-generated forces. However, variations in individual user characteristics made it challenging to provide uniform assistance across all participants, leading to larger tracking errors under reduced guidance conditions. A participant-level summary of the active-assisted mode experimental results from a representative subset of 15 participants is provided in Appendix A Table A3.

## 5. Discussion

### 5.1. Evaluation Results

The representative time responses (Figure 7 and Figure 8) and the tracking-error summary (Table 4) indicate consistent tracking behavior under the tested assistance settings. The integration of a moving-window scheme with guidance force control enables adaptive rehabilitation training through active-assistive control in upper-limb rehabilitation robots. The proposed active control offers adjustable supportive force while maintaining tracking errors within acceptable bounds. The virtual tunnel-based control strategy for the upper-limb rehabilitation exoskeleton demonstrates potential for enhancing rehabilitation outcomes. Torque fluctuations appeared near the virtual tunnel boundaries, particularly when the participant’s arm approached the edges of the moving window, indicating moments when voluntary movements diverged from the target trajectory. Therapist-delivered manual assistance remains essential in assisted rehabilitation, particularly for tailoring tasks and responding to patient-specific needs. The proposed controller is intended to complement clinical practice by providing consistent motion guidance and stable force profiles during device-mediated training. The free space within the virtual tunnel accommodates variations in users’ physical characteristics and guidance needs, enabling stable and personalized motion. Compared with conventional model-based or fixed-impedance approaches, the moving-window scheme adapts the guidance force in response to the user’s engagement and movement within the defined spatial boundary. To further refine the proposed controller’s efficacy, optimizing control parameters to provide supportive forces tailored to the patient’s rehabilitation needs is crucial. Developing patient-centered controllers that adapt to individual capabilities is key to effective and responsive therapy.

### 5.2. Limitations

This study has several limitations. First, validation involved 30 healthy participants under non-therapist-guided, non-resistive conditions. Therefore, the results may not fully reflect the assistance behavior under clinical settings or among individuals with varied motor impairments or training backgrounds. Second, the control law assumes low-speed motion, neglects Coriolis and centrifugal effects, and treats the remaining unmodeled effects as a lumped term, without fully characterizing human–robot interaction disturbances. Third, reference trajectories were generated offline from motion-capture data, with intention detection and adaptive trajectory generation not addressed in this study. Formal statistical significance testing (e.g., ANOVA or *t*-test) will be pursued in follow-up studies with expanded cohorts and datasets to further examine condition-to-condition differences. Practical safeguards include an emergency stop (E-stop) accessible to both the participant and the operator, actuator/driver-level current or torque limiting to prevent excessive interaction forces, and software-based saturation and monitoring (e.g., watchdog mechanisms) to detect abnormal behavior and trigger a safe shutdown. These measures are intended to complement clinical supervision and reduce the risk during device-mediated training. These limitations indicate that future work should: (i) include participants with diverse motor abilities or clinical impairments; (ii) incorporate disturbance observers or interaction-torque estimators with learning-based gain scheduling for guidance force and moving-window size; (iii) characterize frictional and Coriolis effects to enable sensitivity or ablation analyses justifying the modeling simplifications.

## 6. Conclusions

This study validated the effectiveness of the proposed active-assistive control framework integrated with a moving-window strategy for upper-limb rehabilitation. The controller dynamically modulates assistance within a defined spatial boundary, delivering mechanical stimuli that promote motor recovery while encouraging voluntary user participation. A two-DOF wearable exoskeleton targeting wrist and elbow movements was experimentally evaluated in a cohort of 30 healthy participants using two activities of daily living: hand-washing and door-opening. Experimental findings confirmed accurate trajectory tracking under both passive and active-assistive training conditions, verifying the feasibility of the proposed control framework for task-oriented rehabilitation. Furthermore, reductions in the guidance force parameter corresponded to increased tracking errors and decreased peak torques at the elbow and wrist. These findings underscore the controller’s capability to proportionally adjust robotic assistance according to user effort, highlighting its potential to enable patient-centered rehabilitation tailored to individual recovery needs.

## Figures and Tables

**Table 1:** Upper limb rehabilitation robots and controllers.

Authors	Year	DOF	Robot Type	Exercise Mode	Control Methodology
Zhang, D. et al. [14]	2020	6	Exoskeleton	Active-assistive	Fuzzy impedance control
Li, X. et al. [15]	2020	7	Exoskeleton	Active-assistive	Performance-based hybrid control
Zhang, L. et al. [16]	2020	7	End-effector	Active-assistive	Impedance control
Tang, R. et al. [17]	2022	4	End-effector	Active-assistive	Hybrid position/force control
Pareek, S. et al. [18]	2023	6	End-effector	Active-assistive	Reinforcement learning
Zhang, Y. et al. [19]	2024	2	End-effector	Active-assistive	Adaptive impedance control
Yan, Y. et al. [20]	2024	2	Exoskeleton	Passive	Model predictive control

**Table 2:** Modified Denavit–Hartenberg parameters.

*i*	$a_{i-1}$	$\alpha_{i-1}$	$d_{i}$	$\theta_{i}$
1	0	π/2	0	$\theta_{1}$
2	0	π/2	0	$\theta_{2}$
End effector	$l_{3}$	0	$l_{1}+l_{2}$	0

**Table 3:** Participant specifications. This table summarizes participant specifications and ROM ranges relevant to the reference ADL trajectories derived from the N=30 motion-capture dataset.

Age	Subject Number	Arm Length (mm)	Wrist ROM (°)	Elbow ROM (°)
18–25	1–3	520∼540	−77.7∼57.0	0∼75.2
18–25	4–7	541∼560	−79.8∼63.1	−1.1∼79.5
18–25	8–10	561∼580	−77.2∼66.4	−0.9∼78.3
26–30	11–13	520∼540	−71.9∼55.0	−0.1∼75.9
26–30	14–18	541∼560	−75.4∼51.6	−1.1∼75.9
26–30	19–20	561∼580	−77.7∼54.6	−0.7∼78.3
30–35	21–22	520∼540	−51.8∼50.3	−1.6∼77.6
30–35	23–26	541∼560	−53.9∼43.1	−1.1∼79.3
30–35	27–30	561∼580	−59.9∼45.8	−1.9∼79.1

**Table 4:** Tracking error summary in active-assistive mode (unit: °, format: mean ± SD).

Task	$F_{\mathrm{gf}}$	Wrist Error (°)	Elbow Error (°)
Hand-washing	100%	1.58 ± 1.58	1.21 ± 1.17
Hand-washing	70%	2.57 ± 2.54	2.45 ± 1.82
Hand-washing	30%	4.85 ± 4.79	4.09 ± 3.01
Door-opening	100%	0.96 ± 0.96	0.96 ± 0.88
Door-opening	70%	1.73 ± 1.61	2.47 ± 2.29
Door-opening	30%	3.03 ± 2.99	4.81 ± 4.44

## Data Availability

The data presented in this study are available upon request from the corresponding author. The data are not publicly available due to privacy and ethical restrictions involving human participants.

## Appendix A. Participant-Level Experimental Result Tables

**Table A1:** ROM trajectory generation experiment results (30 participants, 2 tasks, unit: °).

Task	Gender	Age	No.	ROM Max/Min		Angle Range per Joint			
				Wrist	Elbow	Wrist Min	Wrist Max	Elbow Min	Elbow Max
Door-opening	Male	20	1	−4.612∼35.815	0.350∼90.571	−4.612	35.815	0.350	90.571
			2	−2.617∼51.997	−0.196∼99.930	−2.617	51.997	−0.196	99.930
			3	−4.287∼44.427	−1.241∼93.765	−4.287	44.427	−1.241	93.765
			4	−4.183∼41.841	−0.449∼99.130	−4.183	41.841	−0.449	99.130
			5	0∼51.097	−1.005∼98.226	0	51.097	−1.005	98.226
			6	3.541∼50.445	−1.582∼96.224	3.541	50.445	−1.582	96.224
			7	−0.944∼44.422	0∼97.909	−0.944	44.422	0	97.909
			8	−8.535∼39.570	−0.142∼100.229	−8.535	39.570	−0.142	100.229
			9	−4.253∼42.808	−1.974∼98.937	−4.253	42.808	−1.974	98.937
			10	−4.195∼46.373	−1.313∼102.160	−4.195	46.373	−1.313	102.160
		30	1	−6.659∼43.572	1.270∼87.146	−6.659	43.572	1.270	87.146
			2	−0.246∼55.063	−0.791∼92.888	−0.246	55.063	−0.791	92.888
			3	−3.007∼38.424	−0.769∼100.919	−3.007	38.424	−0.769	100.919
			4	2.687∼47.334	0∼98.846	2.687	47.334	0	98.846
			5	3.652∼41.843	−0.135∼89.551	3.652	41.843	−0.135	89.551
			6	0∼49.092	−1.156∼95.277	0	49.092	−1.156	95.277
			7	−4.658∼52.065	−2.048∼94.153	−4.658	52.065	−2.048	94.153
			8	−4.690∼40.279	−0.463∼98.734	−4.690	40.279	−0.463	98.734
	Female	20	1	2.770∼35.716	−0.779∼85.732	2.770	35.716	−0.779	85.732
			2	0.906∼46.768	−0.417∼94.110	0.906	46.768	−0.417	94.110
			3	1.818∼47.952	−0.490∼94.897	1.818	47.952	−0.490	94.897
			4	0.338∼43.952	−0.274∼95.959	0.338	43.952	−0.274	95.959
			5	−1.423∼43.745	−0.940∼89.228	−1.423	43.745	−0.940	89.228
			6	−4.641∼46.299	−0.963∼95.211	−4.641	46.299	−0.963	95.211
		30	1	0.625∼37.138	−0.339∼81.943	0.625	37.138	−0.339	81.943
			2	2.757∼46.460	0.077∼73.975	2.757	46.460	0.077	73.975
			3	6.906∼35.877	0.251∼77.079	6.906	35.877	0.251	77.079
			4	−6.459∼38.143	−0.622∼83.160	−6.459	38.143	−0.622	83.160
			5	0∼47.711	−0.749∼94.636	0	47.711	−0.749	94.636
			6	−8.535∼56.803	−0.036∼79.535	−8.535	56.803	−0.036	79.535
Hand−washing	Male	20	1	−33.764∼46.370	−1.708∼43.819	−33.764	46.370	−1.708	43.819
			2	−45.716∼47.842	−0.624∼42.336	−45.716	47.842	−0.624	42.336
			3	−51.381∼48.633	−0.709∼42.776	−51.381	48.633	−0.709	42.776
			4	−41.651∼52.434	0.333∼80.244	−41.651	52.434	0.333	80.244
			5	−72.249∼52.375	0.901∼90.726	−72.249	52.375	0.901	90.726
			6	−76.030∼44.000	0.014∼91.285	−76.030	44.000	0.014	91.285
			7	−77.090∼52.780	−0.019∼98.270	−77.090	52.780	−0.019	98.270
			8	−75.645∼63.871	1.630∼98.415	−75.645	63.871	1.630	98.415
			9	−66.708∼66.419	−0.386∼96.311	−66.708	66.419	−0.386	96.311
			10	−78.688∼62.433	0.290∼95.882	−78.688	62.433	0.290	95.882
		30	1	−44.977∼52.107	−1.593∼35.728	−44.977	52.107	−1.593	35.728
			2	−31.921∼53.433	−0.893∼43.218	−31.921	53.433	−0.893	43.218
			3	−59.964∼55.056	−0.179∼74.874	−59.964	55.056	−0.179	74.874
			4	−59.983∼40.318	1.469∼86.756	−59.983	40.318	1.469	86.756
			5	−66.696∼49.133	−0.372∼96.854	−66.696	49.133	−0.372	96.854
			6	−77.830∼48.127	−0.587∼102.864	−77.830	48.127	−0.587	102.864
			7	−72.321∼53.249	0.262∼98.405	−72.321	53.249	0.262	98.405
			8	−71.020∼60.629	−0.005∼98.715	−71.020	60.629	−0.005	98.715
	Female	20	1	−22.050∼44.659	−0.864∼38.760	−22.050	44.659	−0.864	38.760
			2	−70.629∼35.253	−0.757∼100.488	−70.629	35.253	−0.757	100.488
			3	−48.706∼49.547	−0.647∼98.205	−48.706	49.547	−0.647	98.205
			4	−76.383∼49.057	0.096∼104.400	−76.383	49.057	0.096	104.400
			5	−68.382∼56.046	0.233∼102.449	−68.382	56.046	0.233	102.449
			6	−64.786∼55.727	−0.513∼97.791	−64.786	55.727	−0.513	97.791
		30	1	−30.589∼52.031	−1.208∼43.859	−30.589	52.031	−1.208	43.859
			2	−42.595∼46.752	−1.135∼41.902	−42.595	46.752	−1.135	41.902
			3	−35.742∼58.050	−0.910∼41.399	−35.742	58.050	−0.910	41.399
			4	−41.956∼45.556	−1.760∼42.735	−41.956	45.556	−1.760	42.735
			5	−69.881∼48.181	0.045∼98.163	−69.881	48.181	0.045	98.163
			6	−78.113∼56.031	−0.518∼102.492	−78.113	56.031	−0.518	102.492

**Table A2:** Passive mode experiment results (15 participants, 2 tasks).

Task	Gender	Age	No.	ROM		Max/Min Torque per Joint Range			
				(Unit: °)		(Unit: Nm)			
				Wrist	Elbow	Wrist Min	Wrist Max	Elbow Min	Elbow Max
Door-opening	Male	20	1	−3.962∼39.891	−1.212∼95.803	−24.853	5.383	−13.973	2.796
			2	−3.745∼45.395	−3.541∼105.909	−5.654	6.564	−15.654	6.785
			3	−48.190∼43.187	−6.728∼75.306	−4.838	5.011	−17.475	23.615
			4	−3.174∼42.824	−2.587∼100.127	−5.486	6.854	−11.257	6.518
			5	−2.167∼42.825	−1.485∼105.347	−5.354	5.857	−10.874	5.659
			6	−3.247∼44.378	−1.541∼95.859	−5.725	6.591	−10.254	6.527
		30	1	−4.731∼42.107	−2.04∼102.554	−5.842	6.875	−15.662	6.887
			2	−48.616∼43.172	−9.860∼71.918	−4.665	6.393	−18.262	22.513
	Female	20	1	−3.215∼41.744	0∼99.119	−3.956	6.766	−12.646	4.518
			2	−2.786∼41.711	0∼99.687	−5.328	5.731	−10.466	6.511
			3	−3.165∼41.722	0∼98.325	−4.980	4.698	−10.715	5.159
			4	−2.623∼42.207	−1.002∼99.391	−4.850	5.824	−13.248	6.855
			5	−4.521∼45.847	−1.950∼92.547	−5.652	6.729	−13.224	6.778
		30	1	−3.432∼41.705	−0.118∼95.884	−5.154	5.560	−14.611	3.831
			2	−48.295∼43.077	−10.887∼75.092	−5.702	4.665	−8.659	5.504
Hand−washing	Male	20	1	−49.081∼38.426	−0.511∼74.910	−6.023	10.456	−16.959	2.771
			2	−52.373∼44.609	−3.173∼85.717	−6.054	10.529	−17.245	6.789
			3	−50.973∼44.480	−0.549∼81.611	−6.258	11.469	−16.548	6.892
			4	−52.827∼44.058	−1.154∼80.015	−6.625	11.343	−17.297	6.474
			5	−47.770∼43.218	−0.168∼78.315	−6.758	11.598	−16.787	6.584
			6	−48.253∼43.727	−1.275∼88.477	−6.237	11.647	−15.857	6.458
		30	1	−48.854∼38.391	−0.813∼82.668	−5.817	12.760	−10.439	6.681
			2	−54.275∼43.556	−0.340∼79.513	−6.548	11.215	−17.325	3.584
	Female	20	1	−48.902∼38.384	−1.415∼81.404	−4.985	5.726	−15.665	3.484
			2	−49.106∼38.406	−1.145∼86.959	−6.021	7.010	−14.261	2.492
			3	−47.757∼41.317	0∼81.498	−4.758	6.820	−9.458	6.715
			4	−3.194∼44.427	0∼55.885	−5.529	3.628	−5.353	6.624
			5	−3.765∼44.475	−0.729∼59.247	−5.011	4.147	−5.510	6.939
		30	1	−48.903∼38.408	−0.976∼81.448	−5.682	6.075	−8.235	6.431
			2	−3.344∼44.569	−0.333∼56.977	−6.393	4.147	−8.936	6.679

**Table A3:** Active-assisted mode experiment results (15 participants, 2 tasks).

Task	Gender	Age	No.	ROM		Max/Min Torque per Joint Range			
				(Unit: °)		(Unit: Nm)			
				Wrist	Elbow	Wrist Min	Wrist Max	Elbow Min	Elbow Max
Door-opening	Male	20	1	−1.323∼52.789	−0.004∼108.967	−12.613	8.121	−8.344	8.816
			2	−2.028∼53.031	0∼95.828	−10.194	6.911	−6.927	11.493
			3	0∼45.820	−0.533∼118.813	−7.429	5.011	−10.391	14.641
			4	−38.462∼31.933	−2.108∼88.044	−4.665	5.356	−5.668	11.650
			5	−38.475∼31.995	0∼86.613	−5.011	5.011	−5.195	11.178
			6	−0.475∼32.675	−0.412∼107.19	−4.838	6.393	−7.714	12.437
		30	1	−0.173∼53.538	−1.17∼101.517	−5.183	6.911	−4.251	14.012
			2	−38.478∼31.992	−1.031∼71.449	−5.292	5.356	−5.825	12.437
	Female	20	1	0∼35.192	0∼104.345	−4.147	5.011	−6.455	8.974
			2	0∼42.211	0∼114.236	−5.874	5.024	−5.668	9.603
			3	−12.585∼44.127	−2.166∼102.943	−5.356	6.220	−4.566	8.816
			4	−1.387∼33.967	0∼110.259	−5.365	6.566	−5.825	8.501
			5	−7.420∼42.700	−1.655∼101.420	−6.393	5.874	−6.770	7.557
		30	1	0∼33.817	0∼110.436	−4.319	5.183	−3.621	8.187
			2	0∼34.927	−1.254∼100.246	−4.325	5.259	−3.658	8.654
Hand−washing	Male	20	1	−41.463∼47.709	−0.732∼109.899	−10.021	5.356	−5.510	12.595
			2	−41.306∼64.449	0∼102.014	−5.356	5.529	−5.038	12.497
			3	−41.709∼33.824	−0.783∼106.018	−5.183	6.393	−4.880	12.358
			4	−41.735∼31.500	−0.284∼54.843	−4.838	5.529	−4.251	14.169
			5	47.262∼50.024	−0.525∼92.899	−6.911	5.702	−7.872	15.743
			6	−41.978∼34.693	−0.722∼87.852	−5.011	6.047	−3.464	16.373
		30	1	−41.042∼40.976	0∼99.314	−8.293	5.874	−9.289	11.493
			2	−42.688∼34.106	−1.23∼84.612	−5.702	7.257	−3.464	15.586
	Female	20	1	−33.922∼33.593	0∼96.722	−6.393	6.566	−6.455	9.020
			2	−39.715∼30.652	−0.430∼53.066	−5.356	6.220	−5.825	9.854
			3	−51.495∼44.988	0∼85.152	−4.319	6.911	−5.178	8.187
			4	−41.306∼32.942	0∼102.014	−5.356	5.529	−5.038	9.114
			5	−41.454∼33.822	−0.827∼95.244	−5.183	7.084	−5.029	9.165
		30	1	−52.557∼45.977	0∼67.584	−3.456	5.011	−3.621	7.446
			2	−38.870∼32.084	−1.100∼99.356	−5.293	6.393	−5.825	7.714
